# *Pax6-* and *Six3*-Mediated Induction of Lens Cell Fate in Mouse and Human ES Cells

**DOI:** 10.1371/journal.pone.0115106

**Published:** 2014-12-17

**Authors:** Raymond M. Anchan, Salil A. Lachke, Behzad Gerami-Naini, Jennifer Lindsey, Nicholas Ng, Catherine Naber, Michael Nickerson, Resy Cavallesco, Sheldon Rowan, Jennifer L. Eaton, Qiongchao Xi, Richard L. Maas

**Affiliations:** 1 Division of Genetics, Department of Medicine, Brigham and Women's Hospital and Harvard Medical School, Boston, Massachusetts, 02115, United States of America; 2 Division of Reproductive Endocrinology and Infertility, Department of Obstetrics, Gynecology and Reproductive Medicine, Brigham and Women's Hospital and Harvard Medical School, Boston, Massachusetts, 02115, United States of America; 3 Department of Biological Sciences, Center for Bioinformatics and Computational Biology, University of Delaware, Newark, Delaware, 9716, United States of America; University of Colorado Denver School of Medicine, United States of America

## Abstract

Embryonic stem (ES) cells provide a potentially useful *in vitro* model for the study of *in vivo* tissue differentiation. We used mouse and human ES cells to investigate whether the lens regulatory genes *Pax6* and *Six3* could induce lens cell fate *in vitro*. To help assess the onset of lens differentiation, we derived a new mES cell line (*Pax6*-GFP mES) that expresses a GFP reporter under the control of the *Pax6* P0 promoter and lens ectoderm enhancer. *Pax6* or *Six3* expression vectors were introduced into mES or hES cells by transfection or lentiviral infection and the differentiating ES cells analyzed for lens marker expression. Transfection of mES cells with *Pax6* or *Six3* but not with other genes induced the expression of lens cell markers and up-regulated GFP reporter expression in *Pax6*-GFP mES cells by 3 days post-transfection. By 7 days post-transfection, mES cell cultures exhibited a>10-fold increase over controls in the number of colonies expressing γA-crystallin, a lens fiber cell differentiation marker. RT-PCR and immunostaining revealed induction of additional lens epithelial or fiber cell differentiation markers including Foxe3, Prox1, α- and β-crystallins, and Tdrd7. Moreover, γA-crystallin- or Prox1-expressing lentoid bodies formed by 30 days in culture. In hES cells, *Pax6* or *Six3* lentiviral vectors also induced lens marker expression. mES cells that express lens markers reside close to but are distinct from the Pax6 or Six3 transduced cells, suggesting that the latter induce nearby undifferentiated ES cells to adopt a lens fate by non-cell autonomous mechanisms. In sum, we describe a novel mES cell GFP reporter line that is useful for monitoring induction of lens fate, and demonstrate that *Pax6* or *Six3* is sufficient to induce ES cells to adopt a lens fate, potentially via non-cell autonomous mechanisms. These findings should facilitate investigations of lens development.

## Introduction

The ability to direct ES and induced pluripotent stem (iPS) cell differentiation toward specific tissue fates *in vitro* provides an excellent opportunity to investigate the gene regulatory networks (GRNs) that operate during organ development [1], [2]. While ES and iPS cells hold promise for cell-based therapies, only in a handful of cases is molecular information detailed enough to guide directed differentiation to specific tissue types. The developing vertebrate ocular lens offers a potential system for such approaches, as considerable knowledge exists about the cascade of transcription factors, signaling molecules and cell-cell interactions necessary for head surface ectoderm to develop into a mature optically clear lens [3]–[5]. This process is accompanied by the stepwise specification of the pre-placodal region (PPR) into an anterior sensory placode (ASP) domain and then a pseudostratified ectodermal lens placode. Thereafter, progression through the lens pit and lens vesicle stages occurs, culminating in formation of the lens proper [4]. From this stage on, the lens consists of anteriorly localized cells, termed the anterior epithelium of the lens (AEL), that terminally differentiate into posteriorly localized elongated fiber cells.

Numerous studies demonstrate that lens differentiation involves the action of a conserved GRN that is initiated by a specific set of regulatory genes that includes *Pax6* and *Six3* [5]–[8]. Targeted mis-expression in *Drosophila* of mouse or fly *Pax6* that encodes a conserved paired domain and homeodomain containing transcription factor results in multiple ectopic ommatidial structures on the antenna, wings and halteres [9]. In addition, *Pax6* mis-expression in *Xenopus* results in ectopic eye structures that include lens-like tissue termed “lentoids”, as well as retinal tissue [6]–[8]. The formation of ectopic lentoids in the nasal periocular ectoderm is also noted in mice with conditional deletion of beta-catenin, suggesting that canonical Wnt signaling normally represses lens fate [10]. Thus, repression of canonical Wnt signaling in the surface ectoderm is critical for lens development, and *Pax6* has been demonstrated to directly control expression of several Wnt inhibitors in the presumptive lens ectoderm [11]. Conversely, *Pax6* haploinsufficiency in mice results in the *Small eye* and cataract phenotypes, and nullizygosity results in a failure of lens placode induction and anophthalmia [12]–[17]. Similarly, *PAX6* haploinsufficiency in humans results in the pan-ocular eye disorder aniridia that manifests as cataracts, corneal opacification, and retinal anomalies, while compound heterozygosity for *PAX6* loss-of-function causes anophthalmia [18]–[22]. Thus, *Pax6* appears to function as a key regulatory gene for metazoan eye development, acting as one of several ‘eye specification’ genes that function in an interconnected, non-linear GRN with feedback and autoregulatory circuits.

A second eye specification gene is the *Drosophila* homeobox gene *sine oculis (so)*; its presumptive vertebrate orthologue is *Six3*. Ectopic expression of mouse *Six3* in Medaka fish (*Oryzias latipes*) results in ectopic lentoid formation, presumably by activation of *Pax6* expression in the presumptive lens ectoderm, while *Six3* deficiency in mice results in defective lens induction [8], [23]. Collectively these observations support a key, evolutionarily conserved regulatory function of *Pax6* and *Six3* in metazoan eye development that extends to vertebrate lens induction [24]. Given the conserved role for these two ocular developmental regulators, we hypothesized that ES cells might provide an attractive system to investigate early vertebrate ocular and lens regulatory mechanisms *in vitro*.

Previous studies have shown that both mouse and primate (*Cynomolgus* monkey) ES cells possess the ability to differentiate into lentoids upon prolonged culture *in vitro*. In these studies, the induction of lentoid formation, defined by a characteristic 3-D morphology and the expression of lens markers, involved the upregulation of *Pax6*-expression in differentiating ES cells co-cultured with a stromal cell feeder layer. For example, these cells have been reported to provide stromal cell-derived inducible factors that promote the differentiation of pluripotent stem cells to neuronal pigmented epithelial cell fates [25]–[27].

Two additional reports describe the induction of lens progenitors and lentoids from hES cells and from iPS cells derived from cataract patients using chemically defined protocol [28], [29]. These investigations used a three-step protocol that was based on known signaling requirements in lens development, and achieved efficient induction of lentoid bodies. Collectively, these studies show that ES cells from at least three species - rodent, human, and non-human primate - possess lens forming potential, and suggest a clear role for extrinsic signals in this process. In the case of rodent and non-human primate cells, culture with a stromal feeder layer resulted in increased Pax6 expression in differentiating cells and in the development of lentoid like structures [25]–[27], while in the hES cell protocol, *PAX6* and *SIX3* expression were documented as key early responses in lentoid induction [28], [29]. Given these results, we sought to investigate whether *Pax6* itself, alone or in combination with *Six3*, could directly induce the expression of lens fate in mES and hES cells. We further sought to determine whether this process occurred in a cell autonomous or non-cell autonomous fashion.

## Materials and Methods

### Ethics Statement

All experiments involving derivation of cell lines from any animals was done with the approval of and in accordance with the Harvard University IACUC Approved protocol number 750 (RLM)

### Derivation of *Pax6*-GFP reporter mES cells

**Figure 1:**
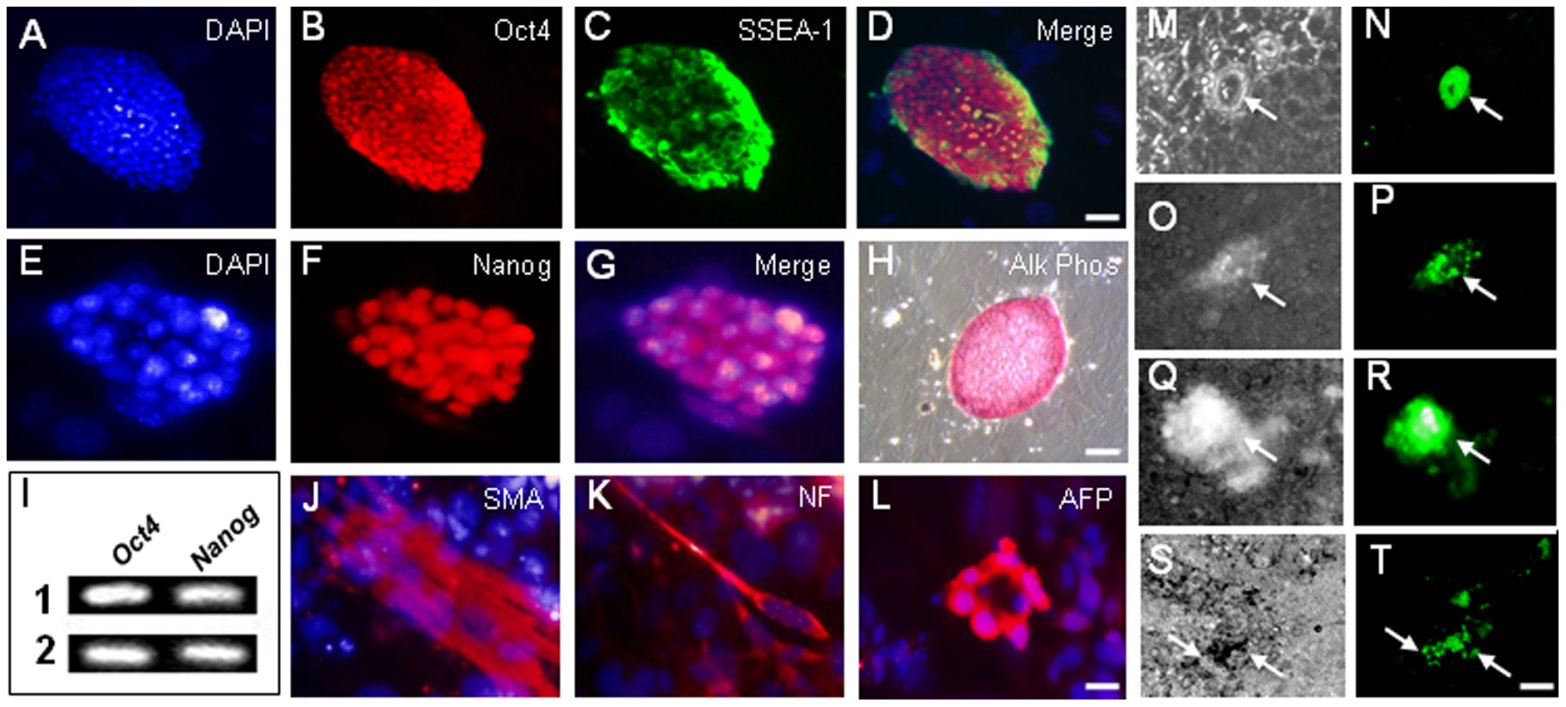
Derivation of a *Pax6-*GFP reporter mES cell line. (**A–H**) Stem cell identity of new ES cell line confirmed by immunofluorscence for undifferentiated ES cell markers Oct4, SSEA-1, Nanog, and by histochemical staining for alkaline phosphatase (Alk Phos) activity. (**I**) *Oct4* and *Nanog* expression confirmed in two *Pax6*-*GFP* mES cell clones by RT-PCR. (**J–L**) Differentiation of mES cells into mesodermal, neuroectodermal and endodermal derivatives confirmed by immunofluorscence for smooth muscle actin (SMA), neurofilament (NF), and alpha-fetoprotein (AFP), respectively. (**M–T**) *Pax6-*GFP reporter expression in mES cells detected following transfection with *Pax6* (**M–O**) or *Six3* (**Q–T**) expression vectors 3 days post-transfection. **M,O,Q,S**, phase contrast; **N,P,R,T** GFP detection. Scale bars: **A-D, H** 40 µm; **E-G, J-L** 10 µm; **M–T** 20 µm.

A novel mES cell line, designated *Pax6*-GFP mES (FVB/N mice) was derived from the previously described transgenic mouse line *Pax6-GFP Cre*, (P0-3.9-GFPCre mice on an FVB background) which expresses GFP reporter under the control of the *Pax6* P0 3.9 ectoderm enhancer (*Pax6 EE*-*GFP*) [30], [31] (S1A **Figure**). All animal studies were conducted in accordance with protocols defined in the ARVO Statement for the Use of Animals in Ophthalmic and Vision Research and approved by the Animal Care and Use Committee of Harvard Medical School (Boston, MA). Blastocysts from these mice were isolated using standard protocols and the inner cell mass was manually separated and cultured with mES cell media on mitotically inactivated mouse embryonic fibroblasts (MEFs). Based on morphologic appearance, presumptive *Pax6*-GFP mES colonies were picked, clonally expanded and characterized by immunohistochemistry, RT-PCR analysis, and differentiation assays, to confirm their stem cell identity (**Fig. 1A–L**).

### Embryonic stem cell and feeder cell sources

Mouse R1 and G4 ES cells were obtained from the Samuel Lunenfeld Research Institute (Mt. Sinai Hospital, University of Toronto, Toronto, Canada). Human H1 ES cells were obtained from the National Stem Cell Bank (WiCell, Madison, WI). MEFs from E13.5 mouse embryos were treated with mitomycin C and used as feeders for mouse G4 ESCs and *Pax6*-GFP mES cells, while gamma-irradiated MEFs were prepared in house or purchased (Global Stem, Inc., Rockville, MD) and used as feeders for H1 hES cells. Feeder preparation and ES cell culture were performed using standard methods [32]–[34].

### *Pax6* and *Six3* expression plasmids and lentiviral vectors

#### Expression plasmids

Mouse *Pax6* or *Six3* cDNAs were cloned into the pcDNA-DEST47 vector using the Invitrogen Gateway Cloning System to produce a CMV promoter-driven C terminal-fused Pax6- or Six3-GFP protein (Invitrogen, Carlsbad, CA). Control cultures were transfected with pBabe Puro expression plasmid (AddGene, Cambridge, MA) originally developed by William Hahn [35] (S1B–C **Figure**).

#### Lentiviral expression vectors

We used both in- house and commercially prepared lentiviral constructs. Commercial constructs were obtained from GeneCopoeia (Rockville, MD; http://www.genecopoeia.com/). Mouse *Pax6* cDNA was cloned into GeneCopoeia ORF clone EX-Mm04345-Lv33 lentiviral vector construct to produce CMV promoter-driven *Pax6* (IRES) GFP proteins. Mouse *Six3* cDNA was cloned into the GeneCopoeia ORF clone EX-Mm05246-Lv43 lentiviral vector construct (S1D–E **Figure**). Additionally, *Pax6* cDNA was cloned into an alternate lentiviral vector HPV570. HPV570 is a self-inactivating, doubly-insulated lentiviral vector that expresses eGFP from an EF1α promoter [36] (a kind gift of Dr. P. Leboulch). *Pax6* cDNA was also cloned into HPV422, a non-insulated lentiviral vector that contains an IRES eGFP cassette and a WPR sequence, and expressed from an *EF1α* promoter [36]. The fragment was cloned between the *Hpa*I and *Mlu*I sites of the lentiviral vector digested with *Bss*HI-*Hpa*I and *Bss*H1-*Mlu*I (S1F–G **Figure**).

### Embryonic stem cell culture, *Pax6* or *Six3* expression vector transfection and transduction

G4 mES cells were cultured on mitomycin C mitotically inactivated MEFs as previously described [32], [33] using DMEM (Gibco, Carlsbad, CA)/10%FBS (HyClone, Logan, UT)/LIF at 10^6^ U/ml (Millipore, Billerica, MA)/0.1 mM b-ME (Sigma-Aldrich, St. Louis, MO) (henceforth defined as ‘mES cell media’). hES cells were cultured on gamma-irradiated MEF feeders in 80% DMEM-F12 (Gibco, Carlsbad, CA)/20% KnockOut Serum Replacement (KOSR, Gibco,)/1 mM L-glutamine (Gibco, Carlsbad, CA)/0.1 mM β-mercaptoethanol (Sigma-Aldrich, St. Louis, MO)/4 ng/ml bFGF (Invitrogen, Carlsbad, CA) (henceforth defined as ‘human ES cell media’). Once ES cell cultures were sub-confluent, these cultures were either transfected with a *Pax6* or *Six3* expression plasmid or infected with the appropriate lentiviral vectors. *Pax6* and *Six3* expression plasmid vectors were transfected into G4 or *Pax6-*GFP mES cells using FuGENE 6 (Roche, Madison, WI). *Pax6* and *Six3* lentiviral vectors were introduced by infecting cells with freshly harvested viral supernatant along with 6 µg/ml of polybrene (Millipore, Billerica, MA). Transfected and transduced ES cells cultures were fixed on days 7, 14, 21, 28 or 30 *in vitro* using 4% paraformaldehyde/4% sucrose at room temperature for 15 minutes. The cultures were then processed for immunostaining as described below.

### Immunostaining of *Pax6* and *Six3* expressing ES cell cultures

After fixation, *Pax6* or *Six3* ES cell cultures were incubated at least 2 hours with a primary antibody for goat polyclonal γA-crystallin (Santa Cruz Biotech., Santa Cruz, CA;), rabbit polyclonal αB-crystallin (Abcam, Cambridge, MA;), rabbit polyclonal Prox1 (Covance; PRB), or rabbit polyclonal Tdrd7 [37] at a dilution of 1∶100. After 3 washes fixed cultures were incubated at least 1 hour in Alexa Fluor 488 or Alexa Fluor 594 (Invitrogen, Carlsbad, CA) conjugated secondary antibody at a dilution of 1∶1000. After immunostaining was completed, cells were incubated for 30 minutes in DAPI to visualize nuclei. For antibody specifications, see S1 **Table**.

### Characterization and immunostaining of the *Pax6*-GFP mES cells

Cultures of *Pax6*-GFP mES cells were fixed in 4% paraformaldehyde (PFA)/4% sucrose and processed for immunostaining. Commercial antibodies for Oct4 (ab18976; Abcam, Cambridge, MA), SSEA1 (FCMAB117P; Millipore, Billerica, MA) and Nanog (ab106465; Abcam, Cambridge, MA), and histochemical reagents for alkaline phosphatase activity (Sigma-Aldrich, St. Louis, MO) were used for marker studies. Differentiation of *Pax6*-GFP mES cells was evaluated by immunostaining with antibodies to neurofilament (NF, ectoderm) (ab24575; Abcam, Cambridge, MA), alpha-fetoprotein (AFP, endoderm) (sc-8108; Santa Cruz Biotech.) and smooth muscle actin (SMA, mesoderm) (ab5694; Abcam, Cambridge, MA). Primary and secondary antibody immunostaining were performed as previously described [38]. Controls included omission of primary or secondary antibody, and comparison of differentiated and undifferentiated cells. For antibody specifications, see S1 **Table**.

### Polymerase chain reaction (PCR)

Total RNA for RT-PCR analyses was isolated from cell cultures using a Qiagen kit (Valencia, CA, USA). RT-PCR analyses were performed using primers for stem cell and lens markers (S2 **Table**). RT was performed using the qScript cDNA Synthesis Kit (Quanta Biosciences, Gaithersburg, MD). The RT product was used in PCR with the GoTaq Core System I (Promega, Fitchburg, WI) for 35 amplification cycles at a 57–60°Celsius annealing temperature.

## Results

### Derivation of a *Pax6*-GFP reporter mES cell line

To genetically monitor if ES cells could be induced to acquire lens progenitor cell fate, we first developed a mES cell line that expresses a GFP reporter under the control of the murine *Pax6* P0 promoter and 3.9 kb of upstream sequence that contains the *Pax6* lens ectoderm enhancer or *EE* [39], [40]. This reporter cell line, denoted *Pax6*-GFP mES, was developed from a mouse transgenic line *Pax6-GFP Cre* [30], [31] that expresses GFP beginning at E8.75 in presumptive lens ectoderm under the control of the *Pax6* P0 promoter and *EE*. We isolated the inner cell mass from blastocysts from this transgenic mouse line and derived ES cells by culturing them in mES cell media on mitotically inactivated mouse embryonic fibroblasts (MEF). Morphologically compact *Pax6*-GFP mES colonies were picked, clonally expanded and characterized by immunostaining, differentiation assays, and RT-PCR analysis to confirm their stem cell identity. Immunostaining demonstrated the expression of the known ES cell markers Oct4, SSEA1 and Nanog (**Fig. 1A–G**). Similar to other ES cell lines, cells in *Pax6*-GFP mES colonies displayed alkaline phosphatase activity, and immunostaining results were verified by RT-PCR detection of *Oct4* and *Nanog* expression in two independent *Pax6*-GFP mES cell line clones (**Fig. 1H–I**).

In addition, when allowed to differentiate, these cells generated mesodermal, ectodermal and endodermal cell types as reflected by immunostaining for smooth muscle actin (SMA), neurofilament (NF) and alpha-fetoprotein (AFP) (**Fig. 1J–L**), respectively. Collectively, these data support the pluripotent capacity of the *Pax6*-GFP mES cell line. Lastly, we found that transfection of *Pax6* **(** **Fig. 1M–O** **)** or *Six3* **(** **Fig. 1Q–T** **)** up-regulated the *Pax6-EE* GFP reporter, as evident from GFP expression as early as 3 days post-transfection (**Fig. 1M–T**). Thus, we derived a *Pax6*-GFP mES cell line that expresses a GFP reporter under the control of the *Pax6 EE*.

### Expression of *Pax6* or *Six3* in mES cells induces lens marker expression

**Figure 2:**
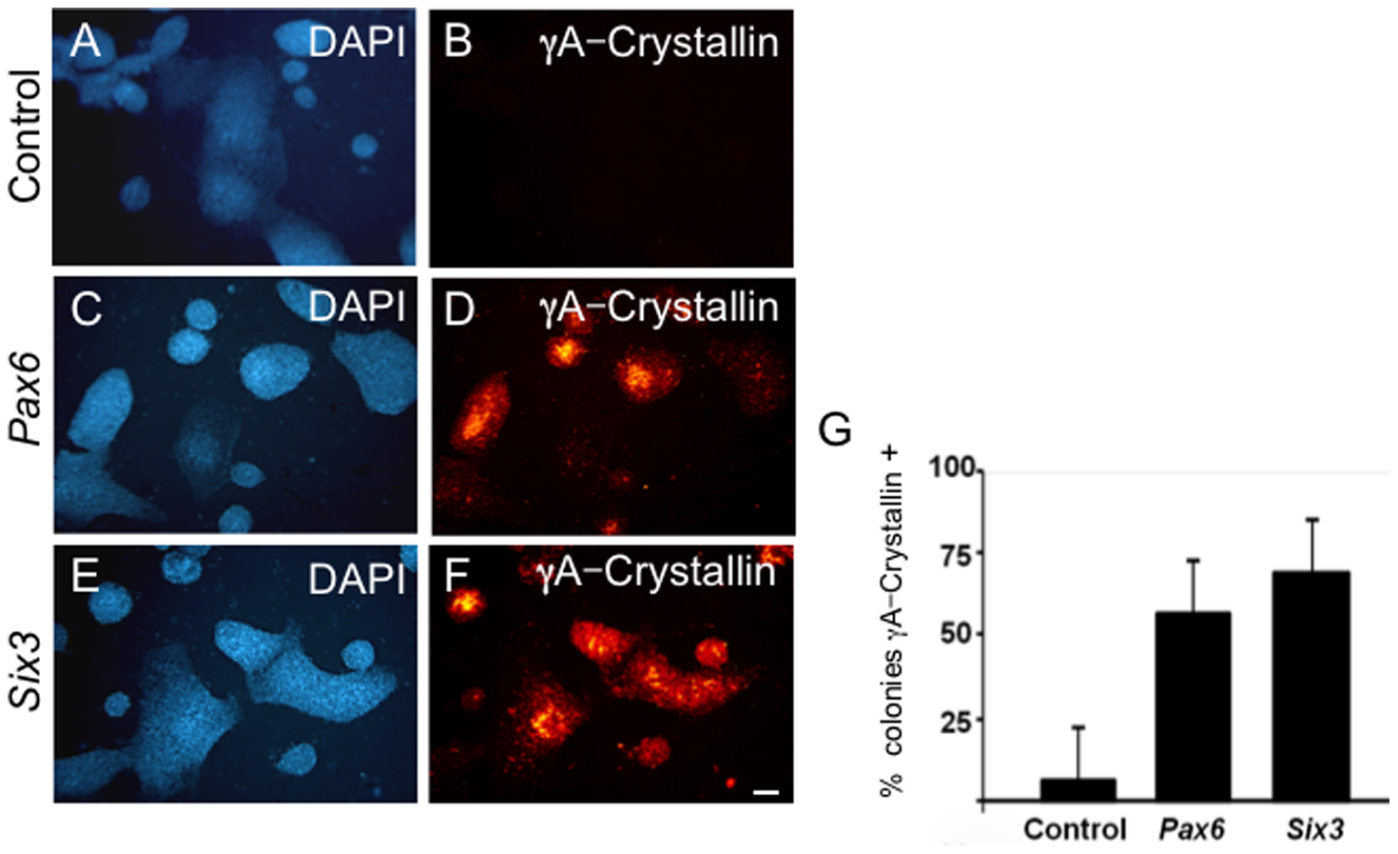
*Pax6* or *Six3* expression in G4 mESC cells induces γA-crystallin expression. **(A–B)** Control mESC cultures transfected with vector alone. (**C–F**) G4 mES cells transfected with (**C–D**) *Pax6* or (**E–F**) *Six3* expression plasmids demonstrate γA-crystallin immunoreactivity by day 7 post-transfection. (**G**) The number of γA-crystallin immunoreactive colonies following *Pax6*- or *Six3*-transfection is>10-fold more than for vector controls. Scale bar: **A–F** 200 µm.

Introduction of *Pax6* or *Six3* into G4 mES cells via expression plasmid resulted in a>10-fold increase in the percentage of γA-crystallin immunoreactive mES cell colonies by 7 days post-treatment compared to G4 mES cells transfected with control plasmids at similar efficiency (**Fig. 2A–G**). Similar results were obtained with lentiviral vector transduction (data not shown).

**Figure 3:**
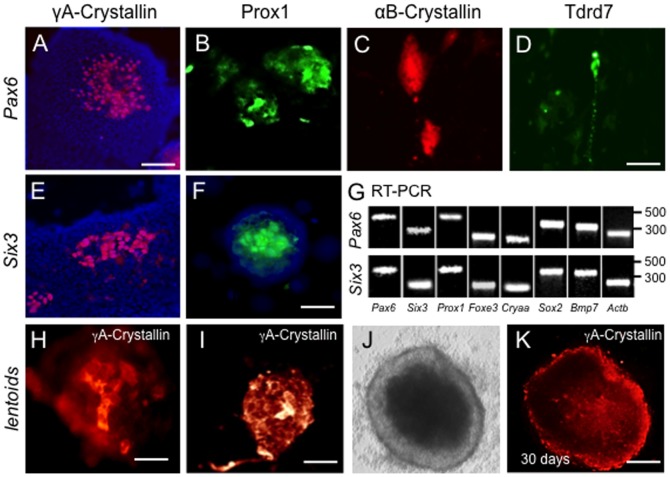
*Pax6* or *Six3* expression in G4 mESC cells induces lens marker expression. (**A–F**) G4 mES cells transfected with either (**A–D**) *Pax6* or (**E,F**) *Six3* expression plasmids exhibit γA-crystallin (**A,E**) and Prox1 (**B,F**) expression at day 7. *Pax6*-transfection also results in expression of (**C**) αB-crystallin, and (**D**) Tdrd7. (**G**) Expression of lens markers in *Pax6*- and *Six3*-transfected G4 mESC colonies confirmed by RT-PCR. (**H–K**) In some cases, γA-crystallin positive mES cells accumulate in aggregates at days 7–14, with further expansion into lentoid bodies at 30 days (**J,** phase; **K,** γA-crystallin immunofluorscence). Scale bars: **A** 75 µm; **B–F** 50 µm; **H–I** 25 µm; **J–K** 50 µm.

After 14 days of culture post-transfection, γA-crystallin expression continued to be detected in these ES cells, mainly in the central portions of individual colonies (**Fig. 3A,E**). We also detected expression of other lens fiber cell differentiation markers, Prox1, αB-crystallin, and Tdrd7 expression by immunostaining in these cultures at this stage whereas control vectors (LvHPV422 and LvHPV570) and vectors encoding either of two other genes, *Eya1*, *Ctnnb* (encoding β-catenin), gave negligible staining (**Fig. 3B–D,F**, and data not shown). These data suggest that *Pax6* and *Six3* can induce markers of lens fiber cell fate in mES cells. Tdrd7 expression was observed to be partly granular (**Fig. 3D**), as previously described in lens fiber cells [37]. RT-PCR analyses confirmed the transcript expression of these and other lens expressed genes (**Fig. 3G**). Thus, expression of either *Pax6* or *Six3* induces the expression of lens fiber cell markers.

In addition, beginning as early as 7–14 days post-transfection of mES cells, distinct cell aggregates of Prox1 (**Fig. 3B,F**) and γA-crystallin expressing cells were observed (**Fig. 3H–J**) at a ratio of ∼4 aggregates per 15 mES cell colonies. By 30 days in culture, much larger 3-dimensional aggregates of compacted γA-crystallin expressing cells could be identified which met the morphological definition of lentoids (**Fig. 3J, K**).

### Human ES cells express lens markers in response to *Pax6* or *Six3* transduction

**Figure 4:**
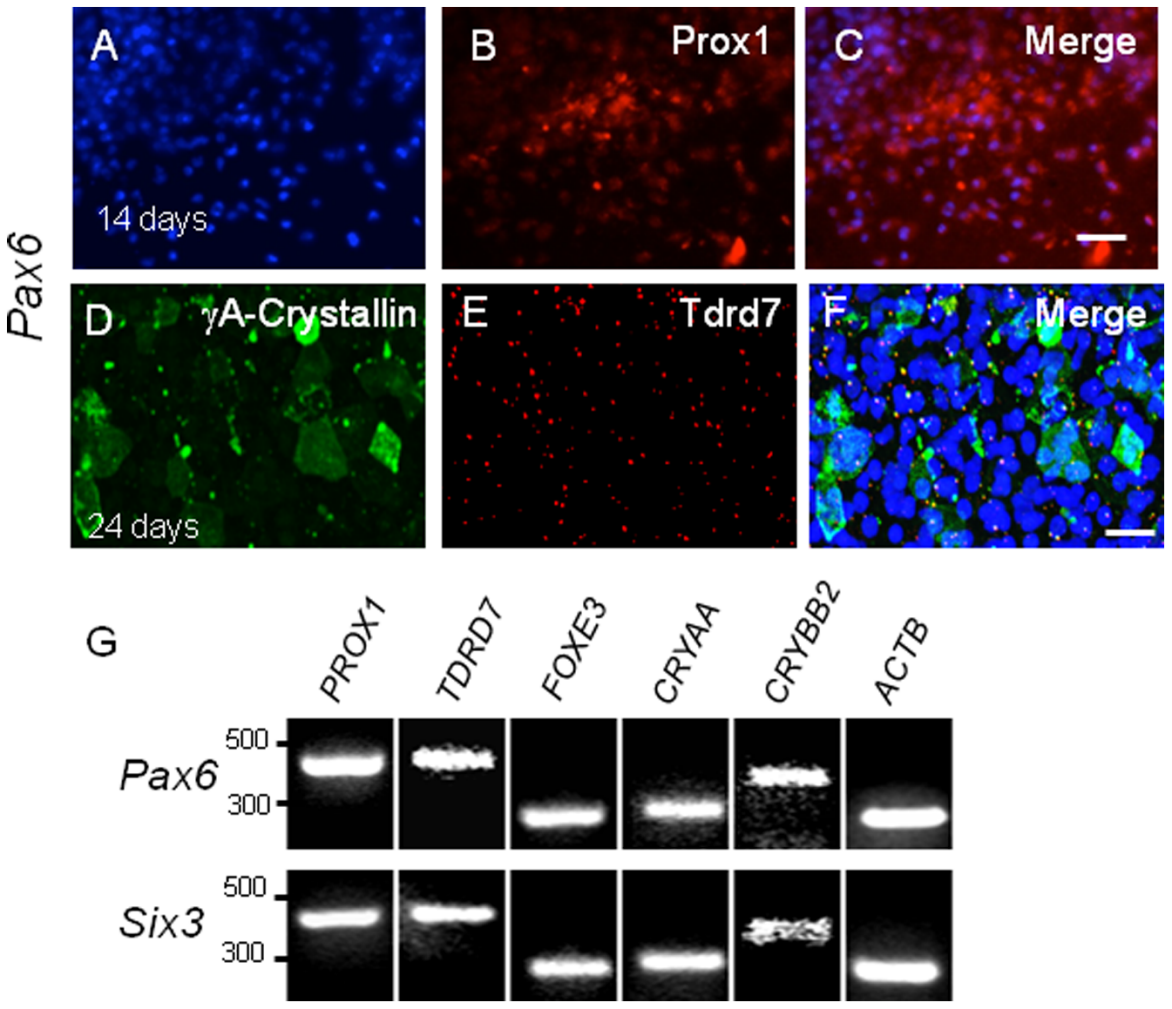
*Pax6* or *Six3* expression in H1 hES cells induces lens marker expression. (**A–F**) H1 hES cells transduced by *Pax6* lentiviral vector express (**A–C**) Prox1 in partly overlapping fashion (**C**) by 14 days post transduction. (**D–F**) By 24 days post-transduction, (**D**) γA-crystallin and (**E**) Tdrd7 are expressed, the latter as cytoplasmic granules. Similar results were obtained following *Six3* transduction (not shown). (**G**) RT-PCR confirms induction of lens marker gene expression in *Pax6-* or *Six3-*transduced H1 hES cells. Scale bars: **A–C** 150 µm; **D–F** 50 µm.

To study whether *Pax6* or *Six3* could induce lens cell fate in human hES cells, we used lentiviral vectors to introduce either mouse *Pax6* or *Six3* into human H1 ES cells. By 14 and 24 days post-infection, *Pax6* lentivirus-infected H1 hES cells exhibited expression of γA-crystallin, Prox1, and Tdrd7 (**Fig. 4A–F**), similar to expression of the homologous mouse proteins in G4 and *Pax6-*GFP mES cells. Prox1 expression was detected in Pax6 transduced hES cells (**Fig. 4A–C**), and so was Tdrd7, which overlapped with a subset of γA-crystallin expressing cells (**Fig. 4D–F**). Note that depending on the type of lens cells Prox1 staining is observed in both nucleus and cytoplasm [41]. In cells of the lens placode, epithelium and the germinative zone, Prox1 is predominantly cytoplasmic, while in differentiating fiber cells it is predominantly nuclear. It is possible that *Pax6* lentivirus-infected H1 hES cells are in the process of differentiation and therefore exhibit staining in both locations. Similar results were obtained with H1 hES cells transduced with a *Six3* expressing lentiviral vector (data not shown), and lens marker results were also confirmed by RT-PCR (**Fig. 4G**).

### ES cell lens differentiation involves distinct cell populations

**Figure 5:**
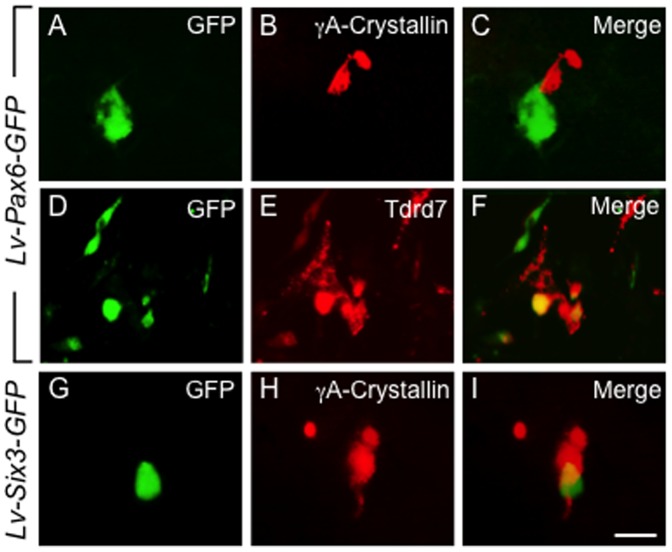
Proximity of lens marker and *Pax6-GFP* or *Six3-GFP* expressing mES cells. (**A–F**) mES cell cultures transduced with *Pax6-GFP* under the constitutive E1a promoter show close proximity but generally non-overlapping expression of GFP with γA-crystallin (**A–C**) or Tdrd7 (**D–F**) at 21 days. (**G–I**) Similar results were obtained for E1a driven *Six3-GFP* transduction and γA-crystallin expression. These results suggest recruitment of undifferentiated mES cells to a lens fate by *Pax6*- or *Six3*-expressing cells. Scale bar: **A–I** 30 µm.

Immunohistochemical analyses of G4 mES cell cultures transduced with *Pax6-* or *Six*3-GFP expressing lentivirus suggested that cells expressing either *Pax6* or *Six3* appeared to induce their neighbors to enter the lens differentiation pathway, but not necessarily themselves. For example, cells that expressed the *Pax6*-GFP vector localized alongside of but were distinct from cells that expressed γA-crystallin and Tdrd7 (**Fig. 5A–F**). Similarly, when *Six3-GFP* was expressed in G4 mES cells at 21 days post-infection, *Six3* GFP expressing cells were often found close to γA-crystallin positive cells, but the two markers only rarely co-localized to the same cell (**Fig. 5G–I**).

## Discussion

The differentiation potential of ES cells makes these cells attractive candidates for cell-based therapies and for unraveling the *in vivo* mechanisms of tissue-specific differentiation. A unique attribute of lens development is the fact that key regulatory genes such as *Pax6* and *Six3* can induce ocular organogenesis in certain invertebrates and vertebrates. In this study we demonstrated that expression of either *Pax6* or *Six3* is sufficient to initiate lens marker expression and lentoid formation in differentiating mouse and human ES cells. By way of comparison, the induction of ES cells to lens fate has also been efficiently achieved by a three step manipulation of signaling pathways known to act in endogenous lens development [28], [29]. Considered together, these complementary results indicate that specific aspects of the endogenous lens forming gene regulatory network (GRN) are recapitulated in the ES cell lens differentiation system.

A novel aspect of the present work was the generation of a mES cell line that expresses a GFP reporter under the control of the *Pax6* P0 promoter and upstream lens ectoderm enhancer (*EE*). This mES cell line should facilitate our understanding of the inductive mechanisms involved in lens progenitor cell differentiation. For example, when *Pax6*-GFP reporter mES cells are transduced with *Pax6* or *Six3,* directed differentiation along the lens pathway appears to commence as early as 3 days post treatment, when GFP reporter expression is detected. *In vivo,* the mouse *Pax6* ectoderm enhancer directs *Pax6* expression as early as E8.5 during lens placode specification and thereafter in the AEL, and it is positively autoregulated by the *Pax6* gene product. Hence, the early appearance of GFP expression following introduction of *Pax6* into *Pax6*-GFP reporter mES cells is consistent with the known positive autoregulation of the *Pax6 EE.* In *Pax6* or *Six3* transfected *Pax6*-GFP or G4 mES cells, differentiation ensues with expression of γA-crystallin and of additional lens differentiation markers. Frequently, these lens marker positive cells were noted to cluster together in aggregates in the central portion of individual ES cell colonies. Ultimately, by 30 days post-transduction, some aggregates coalesce to form lentoid bodies.

The lens marker genes expressed during differentiation in the *in vitro* ES cell system are normally expressed in distinct spatial and temporal patterns during *in vivo* lens development. Specifically, the developing lens involves a single progenitor cell lineage with multiple states of differentiation. Therefore, the significant degree of non-overlapping expression of lens markers in differentiating ES cells may reflect the emergence of distinct lens cell phenotypes via normal developmental regulatory mechanisms. Alternatively, the discordant expression of the lens markers in differentiating cells in these cultures could reflect a high degree of cellular and molecular heterogeneity due to variable micro-environmental cues, nor are these two mutually exclusive. Both early markers (*Cryaa*, *Foxe3/FOXE3*, *CRYAA* and *CRYBB2)* as well as late markers of lens cell development (γA-crystallin, Tdrd7, Prox 1), are identified and described in mESC and hESC cultures using immunolabeling while concurrently demonstrating up regulation of *Pax6* expression in both *Pax6* and *Six3* transduced ES cultures. These findings further lend support to the recapitulation of physiologically relevant differentiation pathways *in vitro*. It is important to note that the overall efficiency of lens induction in these cultures appears to be less than that observed in the chemically defined media protocol (27). This is not unexpected, because in the *Pax6* transduction protocol described here, only a fraction of cells are transduced, whereas in the chemically defined media protocol, the entire culture is uniformly exposed to the requisite signaling molecules. Nonetheless, our observations indicate that expression of *Pax6* or *Six3* in undifferentiated ES cells is sufficient to direct a subset of the cells to differentiate towards a lens fate.

These findings hold relevance for two reasons. First, this system may allow the study of lens differentiation mechanisms *in vitro*. Such knowledge could help delineate the underlying genetic circuitry used in endogenous lens development and also needed to generate lens cells from undifferentiated ES cells for future cell-based therapies. Second, an *in vitro* model for lens development could allow study of the pathological mechanisms that underlie congenital lens defects. For example, recently Lachke *et al.* [37] found that mutations in the gene encoding the RNA granule protein Tdrd7 cause cataracts and an associated glaucoma. The presence of Tdrd7 granules in these cultures provides a potential system to further analyze their composition and function. In addition, this system could allow functional tests of lens associated candidate genes identified by bioinformatics tools such as *iSyTE* [42].

Mechanistically, the idea that a single-gene manipulation can initiate the development of a complex tissue is highly appealing and can be understood in the context of scale free networks in which certain highly connected nodes function as “hubs” (41). In this case, key upstream regulatory genes such as *Pax6* and *Six3* may function as hubs and serve to initiate a series of distinct downstream transcriptional events and cellular interactions that lead to the emergence of lens cell types. Previous studies have shown that co-culture of primate and mouse ES cells on PA6 stromal feeders can direct ES cell differentiation along the lens pathway, the latter in a *Pax6*-dependent process [25]–[29]. These results suggest an important role for signaling interactions between feeder and ES cells. An important role for signaling interactions is also indicated by the efficient induction of lens cell fate in chemically defined ES cell induction protocols (27–28).

By tracing the mES cells transduced with a lentiviral vector constitutively expressing either *Pax6* or *Six3* along with GFP under a constitutive EF1α promoter, we were able to track the fate and location of the *Pax6* or *Six3*-expressing cells relative to the lens marker expressing cells in these cultures. Interestingly, we found that while ∼1–5% of GFP expressing cells co-express lens markers, the majority of lens-marker-expressing cells reside near *Pax6-*GFP expressing cells. This observation is consistent with results from the aforementioned co-culture experiments, as *Pax6* expressing cells appear able to recruit nearby undifferentiated cells into the lens differentiation program. We therefore suspect that individual *Pax6* expressing cells recruit other cells to the lens pathway via non-cell autonomous mechanisms, and that the expression of *Pax6* suffices to initiate this differentiation cascade. We also have investigated the use of FGFs which supported formation of lentoids but did not appear significantly different from Pax6 and Six3 transfected differentiating cultures that were not supplemented with FGF. This may in part be due to paracrine production of FGF in all cultures. This system thus provides the opportunity to further investigate the gene regulatory mechanisms that underlie mammalian lens development.

## Supporting Information

S1 Figure **(A–G) Plasmid and viral vector maps and (H) target cells.**
(TIFF)

S1 Table **Antibodies used for immunolabeling in this study.**
(DOCX)

S2 Table **List of PCR primers used for analysis.**
(DOCX)
