# Supplementary figures and images for: Pax6- and Six3-Mediated Induction of Lens Cell Fate in Mouse and Human ES Cells
Source: PLoS One. 2014 Dec 17;9(12):e115106. doi: 10.1371/journal.pone.0115106 (PMC4269389; doi:10.1371/journal.pone.0115106)

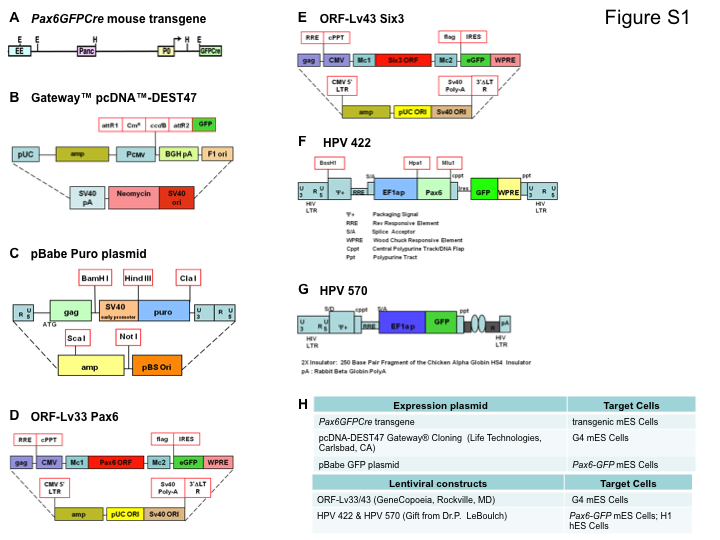

Supplement: S1 Figure — (A–G) Plasmid and viral vector maps and (H) target cells. (TIFF) [file pone.0115106.s001.tif]
