# Supplementary material for: Pax6- and Six3-Mediated Induction of Lens Cell Fate in Mouse and Human ES Cells
Source: PLoS One. 2014 Dec 17;9(12):e115106. doi: 10.1371/journal.pone.0115106 (PMC4269389; doi:10.1371/journal.pone.0115106)
Supplement: S1 Table — Antibodies used for immunolabeling in this study. (DOCX) [file pone.0115106.s002.docx]

**Table S1: Antibody reagents employed**

| **Protein** | **Antibody** | **Vendor** |
| --- | --- | --- |
| PAX6 | Goat polyclonal (sc-7750) | Santa Cruz, Santa Cruz, CA |
| SIX3 | Goat polyclonal (sc-49114) | Santa Cruz, Santa Cruz, CA |
| SSEA1 | Mouse monoclonal (FCMAB117P) | Millipore, Billerica, MA |
| SSEA4 | Mouse monoclonal (FCMAB116P) | Millipore, Billerica, MA |
| FOXE3 | Rabbit polyclonal (ab72596) | Abcam, Cambridge, MA |
| PROX-1 | Rabbit polyclonal (PRB-238C) | Covance, Princeton, NJ |
| αA CRYSTALLIN | Rabbit polyclonal (sc-22743) | Santa Cruz, Santa Cruz, CA |
| αB CRYSTALLIN | Rabbit polyclonal (ab13497) | Abcam, Cambridge, MA |
| βB1 CRYSTALLIN | Mouse monoclonal (ab54655) | Abcam, Cambridge, MA |
| γA CRYSTALLIN | Goat polyclonal (sc-82603) | Santa Cruz, Santa Cruz CA |
| TDRD7 | Rabbit polyclonal | Lachke et al. 2011 |
| STAU1 | Rabbit polyclonal (ab73478) | Abcam, Cambridge, MA |
| OCT4 | Rabbit polyclonal (ab18976) | Abcam, Cambridge, MA |
| NANOG | Rabbit polyclonal (ab106465) | Abcam, Cambridge, MA |
| SMOOTH MUSCLE ACTIN | Rabbit polyclonal (ab5694) | Abcam, Cambridge, MA |
| NEUROFILAMENT | Mouse monoclonal (ab24575) | Abcam, Cambridge, MA |
| α FETOPROTEIN | Goat polyclonal (sc-8108) | Santa Cruz, Santa Cruz, CA |
