# Supplementary material for: Pax6- and Six3-Mediated Induction of Lens Cell Fate in Mouse and Human ES Cells
Source: PLoS One. 2014 Dec 17;9(12):e115106. doi: 10.1371/journal.pone.0115106 (PMC4269389; doi:10.1371/journal.pone.0115106)
Supplement: S2 Table — List of PCR primers used for analysis. (DOCX) [file pone.0115106.s003.docx]

**Table S2: PCR primer pairs employed**

| **Primer** | **Amplicon**  **Size (bp)** | **Sequence** |
| --- | --- | --- |
| Pax6  Forward  Reverse | 410 bp | 5’-CGCTGACTTTCGGTTCTTTC-3’  5’-CTCTTGGGTCAGCTCAGTCC-3’ |
| Six3  Forward  Reverse | 325 bp | 5’-CCACTGCTCCCTACTTCTGG-3’  5’-CGACTCGTGTTTGTTGATGG-3’ |
| Foxe3  Forward  Reverse | 273 bp | 5’-GAAGCCGCCCTACTCATACA-3’  5’-AGGAAGCTACCGTTGTCGAA-3’ |
| Prox1  Forward  Reverse | 435 bp | 5’-CAGCCCGAAAAGAACAGAAG-3’  5’-AGACTTTGACCACCGTGTCC-3’ |
| Cryaa  Forward  Reverse | 203 bp | 5’-CATTCAGCATCCTTGGTTCA-3’  5’-CAAACTTGTCCCGGTCAGAT-3’ |
| Bmp7  Forward  Reverse | 394 bp | 5’-GGGCTTACAGCTCTCTGTGG-3’  5’-GGTGGCGTTCATGTAGGACT-3’ |
| Sox2  Forward  Reverse | 466 bp | 5’-AGAACCCCAAGATGCACAAC-3’  5’-ATGTAGGTCTGCGAGCTGGT-3’ |
| Oct4 (Pou5f1)  Forward  Reverse | 224 bp | 5’-TCTTTCCACCAGGCCCCCGGCTC-3’  5’-TGCGGGCGGACATGGGGAGATCC-3’ |
| Nanog  Forward  Reverse | 364 bp | 5’-AGGGTCTGCTACTGAGATGCTCTG-3’  5’-CAACCACTGGTTTTTCTGCCACCG-3’ |
